# Educated Ability and Industry Essential to Success in the Practice of Dentistry as a Specialty

**Published:** 1887-09

**Authors:** J. H. Grant


ARTICLE III.

### EDUCATED ABILITY AND INDUSTRY ESSENTIAL TO SUCCESS IN THE PRACTICE OF DENTISTRY AS A SPECIALTY.

BY J. H. GRANT.

It has been well said that no physician is qualified to practice a specialty in medicine or surgery who is not well informed upon the fundamental branches as well as the general practice of Medicine. The same opinion will, I think, apply to the dentist, who is, in reality, but practicing a special branch of the great healing art. No man can have too much knowledge of his profession. The world owes its progress to the man of thought, and the man of action. When both are united in the same person, the result is more than doubled, and the value of the service is largely increased. I cannot appeal too strongly for a general intelligence, which is the soil out of which the best specialists grow. It may be said that some of the great men of the world have had but little opportunity for the training of the schools, and yet they have attained distinction as public benefactors; but they are generally recognized as exceptions to the rule, and they for that reason alone are brought before the public more prominently.

Go back fifty years ago; yes, go back twenty-five years ago, and compare dentistry then with the science of it to-day, and what remarkable progress do we find. And I venture to say there is no profession in which there has been greater advancement in the same length of time than you find in the science of dentistry. And this progress is seen not only in what has been attained in the principles and practice of the profession, but also in the character and standing of its members. Many of you can remember the time when the populace thought to be a dentist required no higher talent or skill than that of a barber or blacksmith.

But what changes do we find to-day. We find it risen to that degree of proficiency that it has become a respectable profession, which to be successfully followed demands long study and thorough training, with an amount of scientific knowledge and practical skill equal to that of any other profession.

But we must confess that there are to-day, in the dental profession, as there are in other professions, those who are sadly deficient in the qualities and qualifications that are necessary to give stability to the character and standing of members of our profession. How are we to remedy this? The only way that I can see for it to be done, is to compel every one whose application comes before our examining board to either present a certificate of identify with the profession, or a diploma from some reputable school as a graduate, or pass a rigid examination before a board of educated practical dentists, in anatomy, physiology, pathology and surgery, indicating an examination entitling those who can creditably sustain themselves therein to true professional rank. I would not be arrogant or exclusive. It is desirable and inevitable, and there will always be a wide difference in the standing and attainments of dentists. I would recognize the humblest degree of skill that can recommend itself to be practical and useful. Hence I would give the hand of welcome to those young men who enter our profession that are ambitious, and by study and perseverance have qualified themselves to pass a searching and satisfactory examination without the aid of schools, and who are entitled to take rank in our profession equally with those who have been more favored by circumstances, and have availed themselves of the important, and to many the necessary aid which the schools afford. If we use discretion in our Association in admitting candidates, and the members are equally particular as preceptors in qualifiying students, we will have no fears that it will be the best and only means of inducing those who enter the practice always to take a pride in elevating the profession.

To be thoroughly educated, practical and scientific means success; without this you cannot hope to succeed. There is a greater demand than ever before, upon the profession, for a higher order of acquaintance with different pathological conditions, and particularly in correct diagnosis. Hence the responsibility rests on us to strive to bring into the profession men of ability, and improve, if possible, those who are already among us, to a degree of proficiency in literature and science that will enable their fingers to work out the thoughts of their brains.

But in order to achieve success in any specialty, a general educaiion, coupled with a knowledge of the science of that specialty, is absolutely essential to the successful practice of that specialty; hence we usually find that those who are specialists, are generally those men who have in time been general practitioners. So in the practice of dentistry as a specialty. The young man who has just returned from college after having graduated is apt to relax his studies and to imagine that he has reached the goal of his dental ambition, and only sits supinely down to await the great reputation that he anticipated during his college course.

Most of those who receive the degree of D. D. S. are at best only prepared to effectively begin the study of their chosen profession. In fact it is only then that they realize the importance of closer application to their studies in order to overcome the sensibility of their ignorance. Having first attained that proficiency, they are better able to concentrate their best energies in the exploration of the vast field of dentistry.

And now, how are we to attain the success in the practice of dentistry, after thoroughly qualifiying ourselves for the practice of that specialty? Are we only to get a good location in some thriving town, among good, thrifty-going people, look out a good office location, fix it up in handsome style, put out a large sign with your name on it, appear in the first issue of your daily newspaper with a card announcing that Dr. So-and-so has arrived with all the latest improvements, (without the ability) known to the profession, and call particular attention to the cheapness of work—low prices—all done in the best manner, and can only be done by him; and sit down in your office and wait for them to come. Well, sometimes it looks as if that were about all for some classes of men to do, for they succeed oftentimes while honest, practical, scientific men do not.

There are some who, it seems are foreordained to fail, while there are others who seem foreordained to succeed.

Whatever one man attempts turns to a failure; whatever another man touches proves to be a success. One man fishes in a stream all day without catching any fish; another man goes to the same stream and at the same place and catches more than he can carry home. Our good friend whom we all respect goes to congress, and gives promise of becoming a great orator and statesman, but in a short time we hear no more of him. The Rev. Mr. S—— closes his course of study and is ordained, but there is no place for him in the vine-yard. Dr. T—— opens an office; tries to make a living by a legitimate practice, but fails, and soon closes up and seeks out a new field for labor, or goes into some other business. And so it goes.

It is a sad thing to fail; but there is no recipe which will positively guarantee a continued success in any business, except qualification, ambition, and a never-ceasing energy, coupled with a condition of our compliance to good and faithful work.

A radical mistake in the choice of a profession generally proves fatal. A man ought to find out what he is capacitated for before he starts. If he enters upon a vocation that is above his capacity he is sure to fail. No amount of industry can supply the natural defect. Some people will tell you that if you will only stick to it, no matter what line of life you have chosen, you are certain to succeed in the end. Many a poor, disappointed dentist will tell you this is not true; he has done his best and failed.

In every profession there is a large percentage of failures, simply because there are so many who strike higher than they can reach. It requires no great amount of foresight to predict that certain persons whom we know can never be artists, or poets, or public speakers, and it requires but a momemt's notice, oftentime, to the qualified experienced and ambitious dentist, to tell who are his successful competitors in his profession. If one has the qualifications and genius for any particular profession, it will manifest itself soon enough. If the fire is in him it will soon blaze out of itself. Ambition is the spur that makes men struggle with destiny. It is life's own incentive to make purpose great and achievement greater.

Each of us has his special label for a particular position, and by it we steadily and regularly, even though sometimes struggling against it ourselves, or though struggled against by others, fall into our appropriate places. Neither can we attribute this all to a fatal necessity. We are not blind-folded, shackled, manacled and led captive by an inexorable fate, or irresistible power, except as our condition is necessarily determined by previous causes, which are themselves mostly in our power, or in the power of those who have the controlling influence of our early years, frought with the conditions of a future necessity.

The world generally pushes a man the way he makes up his mind to go. If going upward is his motto, it pushes him up. If going downward, it pushes him down, and that very rapidly, gravitation always supporting the decline.

But men, if they are qualified, and have that merit that is necessary to sustain their chosen profession, will at last, through perseverance, reach the goal of their ambition to which they are entitled; and those who are not qualified and have not the merit will be ejected with contempt and derision.

But it is no small evil, that the avenues to fame should be blocked up by a swarm of noisy, pushing, elbowing pretenders, who, though they will not ultimately be able to enter themselves, hinder others who have by their perseverance and industry a right to enter.

Some men of talent, however, turn away in disgust from pursuits in which success appears to bear no proportion to defeat. Yet there are some who have sufficient confidence in their own talents, and sufficient elevation of mind to wait with patience while dunce after dunce presses before them.

But let me admonish the younger members of this fact:

that success is impossible without labor also. A fortune is not made in a day, without toil, neither is a reputation made without qualifications and industry. Newton said that "all he had ever accomplished was the result of industry," but it was his genius that made him industrious. So with us in our profession: ability and willingness to labor are the two great conditions of success. "And the degree of estimation in which any profession is held, becomes the standard of the estimation in which the professors hold themselves."—[Burke.]

DISCUSSION.

Dr. Staples: *Mr. President and Gentlemen*—I don't feel like we ought to allow that paper to pass without discussion. Some points in it are worthy the consideration of any man; some things which I have said a good deal about. There is no use talking about making a dentist of a man that nature has not predestined to be such. If we as dentists would properly consider this matter in taking students into our offices there would be fewer failures in the profession than there are, and until we do this thing failures will multiply every day. That we can't make a dentist out of every young man I become more thoroughly convinced every day. Yet men enter the dental profession and make failures and never seem to comprehend why it is they fail, while to me it is perfectly plain. I believe I can plug a tooth, but take no credit to myself for it; I was born that way. The idea seems to prevail among practitioners that any young man of average sense can be made a first-class dentist. It cant be did. I am satisfied that is the cause of half the failures Dr. Grant speaks of in his paper. In the selection of students we should be exceedingly particular. In a practice of twenty years, and applications to the number of fifty during that time, yet I have my first student to take. The reason I have never accepted one was because I did not think they were the proper material to make dentists; and of all I have refused I don't think one is a dentist to-day.

Dr. Williams: I agree with Dr. Staples to a certain extent. A man must possess the necessary natural endowments, yet while quite small at first, by perseverance and cultivation may be developed and finally make a first-class dentist and a success. We are not all perfect by any means, and we are not all born thorough dentists.

Dr. Turner: *Mr. President*—I wish to endorse what Dr. Staples has said. I was much impressed with what I saw in a paper saying that "Dentists are born, not made." I can say "Amen" to that. Unless a man has music in his soul you can never make him a musician. He may get so he can hammer a tune, but not much music. Just so in the mechanical department of the dental art. I believe it is a talent that is born in a man, just like music, drawing oratory, or anything else, and if we would be more careful in selecting material as students it would tend to elevate the profession to a greater degree of success.

Dr. Barton: I endorse those things which were said. A prominent lady was asked why she cut her hair short, and she said it was because she was born with short hair; and I think we were born with a capacity for dentistry that must be cultivated. A few persons have no qualifications for this kind of pursuits, but the best portion of those qualifications for dentistry is acquired. Some of it is born with them.

Dr. J. H. Grant; *Mr. Chairman*—I have had no experience with students, because I have always been a student myself. But while at college I had the opportunity of seeing a great deal of raw material develop into finer material. Students who came there perfectly green, not knowing anything about the mechanism used in the profession, and while they were looked upon as students who would never accomplish any great results, yet by proper instruction would turn out to be very bright lights—superior to many who had had better advantages.

Dr. Staples: *Mr. President*—I am satisfied if the history of those men who attained success could have been learned it would have been found that they were natural mechanics. I have heard a great deal about failures in materials. One man advocates amalgam, another tin foil, another soft gold foil, and this, that and the other; and the only difficulty is that there is not a natural mechanic at the end of the instrument to apply the material to the tooth. There is no doubt but some failures are the result of a want of thoroughness in the operation, but a majority of them are for want of natural skill when they fail in dentistry, and if you sift the thing to the bottom you will find that the good dentists were natural mechanics from the start.

Dr. Goolsbie; *Mr. President*—I think we would be justified in going a little back to the position the gentlemen have taken. I have been watching a little history myself—hereditary predisposition. I think there is a great deal in it, and it is our duty as dentists, if we wish to promote the best interests, and elevate the standard of the profession, to be very careful in selecting our students.

Dr. Storey; There are some facts presentable for our consideration in the varied business and professional pursuits of life which we cannot ignore. We see a man start out in life with a fine education, seemingly qualified for any pursuit. He attempts to merchandise and makes a failure. Another man starts out with equally fair prospects; he has been educated for a doctor. He leaves college bright in theory; full of hope, and returns home and opens a nice office; puts himself behind a two-forty horse—and makes a failure as a physician. The reason for this is, because the Almighty never intended him to be a physician. It is the duty of parents to find the bent of the child's disposition, and to cultivate it. A child always starts in the right direction, and if you, as a parent, will encourage and cultivate his inclinations, he will be sure to "get there."—*Texas Dental Journal.*

